# Berberine Attenuates Acetamiprid Exposure-Induced Mitochondrial Dysfunction and Apoptosis in Rats via Regulating the Antioxidant Defense System

**DOI:** 10.3390/jox14030061

**Published:** 2024-08-07

**Authors:** Annu Phogat, Jagjeet Singh, Reena Sheoran, Arun Hasanpuri, Aakash Chaudhary, Shakti Bhardwaj, Sandeep Antil, Vijay Kumar, Chandra Prakash, Vinay Malik

**Affiliations:** 1 Department of Zoology, Maharshi Dayanand University, Rohtak 124001, India; annu.rs.zoo@mdurohtak.ac.in (A.P.); jagjeet.rs.zoo@mdurohtak.ac.in (J.S.); reena.rs.zoo@mdurohtak.ac.in (R.S.); arun.rs.zoo@mdurohtak.ac.in (A.H.); aakash.rs.zoo@mdurohtak.ac.in (A.C.); shakti.rs.zoo@mdurohtak.ac.in (S.B.); 2 Department of Biochemistry, Maharshi Dayanand University, Rohtak 124001, India; vksiwach.biochem@mdurohtak.ac.in; 3 Department of Zoology, ANDC College, University of Delhi, New Delhi 110019, India; sandeep@andc.du.ac.in; 4 School of Life Sciences, Jawaharlal Nehru University, New Delhi 110067, India

**Keywords:** acetamiprid, berberine, apoptosis, antioxidants, mitochondria

## Abstract

Acetamiprid (ACMP) is a neonicotinoid insecticide that poses a significant threat to the environment and mankind. Oxidative stress and mitochondrial dysfunction are considered prime contributors to ACMP-induced toxic effects. Meanwhile, berberine (BBR) a natural plant alkaloid, is a topic of interest because of its therapeutic and prophylactic actions. Therefore, this study evaluated the effects of BBR on ACMP-mediated alterations in mitochondrial functions and apoptosis in rat liver tissue. Male Wistar rats were divided into four groups: (I) control, (II) BBR-treated, (III) ACMP-exposed, and (IV) BBR+ACMP co-treated groups. The doses of BBR (150 mg/kg b.wt) and ACMP (1/10 of LD_50,_ i.e., 21.7 mg/kg b.wt) were given intragastrically for 21 consecutive days. The results showed that the administration of ACMP diminished mitochondrial complex activity, downregulated complex I (ND1 and ND2) and complex IV (COX1 and COX4) subunit mRNA expression, depleted the antioxidant defense system, and induced apoptosis in rat liver. BBR pre-treatment significantly attenuated ACMP-induced mitochondrial dysfunction by maintaining mitochondrial complex activity and upregulating ND1, ND2, COX1, and COX4 mRNA expression. BBR reversed ACMP-mediated apoptosis by diminishing Bax and caspase-3 and increasing the Bcl-2 protein level. BBR also improved the mitochondrial antioxidant defense system by upregulating mRNA expression of PGC-1α, MnSOD, and UCP-2 in rat liver tissue. This study is the first to evaluate the protective potential of BBR against pesticide-induced mitochondrial dysfunction in liver tissue. In conclusion, BBR offers protection against ACMP-induced impairment in mitochondrial functions by maintaining the antioxidant level and modulating the apoptotic cascade.

## 1. Introduction

Acetamiprid (ACMP) is a broadly used, systemic neonicotinoid pesticide intended to control insect pests due to its fast action and low cost. Consistent use of ACMP in agriculture is responsible for its accumulation in water resources [1] and soil [2]; it has also been detected in several food products [3]. Epidemiological studies have reported the presence of ACMP and its metabolites in the urine of infants [4], children of the three-year age group [5], and in the urine of farmers [6], highlighting the need to evaluate the deleterious effects of ACMP on human health. The clinical signs of acute acetamiprid pesticide poisoning in humans were comparable to those of acute organophosphate intoxication [7]. A case report has documented the human casualty of the accidental intake of ACMP [8]. In mammals, exposure to ACMP is known to cause hepatotoxicity, neurotoxicity, reproductive toxicity, and several health complications [9,10,11,12]. An accumulating body of evidence reported an imbalance of free radical generation, alterations in the intrinsic antioxidant system, biomolecular damage, and structural changes, indicating oxidative stress as a lynchpin for ACMP toxicity.

Mitochondria are prime organelles responsible for metabolism and energy production. Studies have shown that mitochondria are closely associated with oxidative stress within cells as impairment in mitochondrial functions increases reactive oxygen species (ROS) generated by electron leakage from the electron transport chain (ETC) [13,14,15]. Complexes I, II, and IV are principal constituents of ETC, and studies have provided compelling evidence that xenobiotics impair mitochondrial complex activity, resulting in oxidative stress and mitochondrial dysfunction. Impaired complex activity transforms oxygen into superoxide radicals, which further form hydroxyl radicals, depleting the mitochondrial antioxidant defense system [16,17,18]. Mitochondrial superoxide dismutase (MnSOD) and uncoupling protein-2 (UCP-2) are prime proteins implicated with limiting the ROS production in mitochondria by quenching the superoxide radical and uncoupling the oxidative phosphorylation [19,20,21]. Reports have shown that disruption of MnSOD may cause apoptosis, while its optimum expression attenuates apoptosis and maintains mitochondrial functions [22]. Inside mitochondria, ACMP exposure has been reported to disturb redox status, disrupt mitochondria integrity, and downregulate ATP and cAMP production in rat tissues [23,24].

Oxidative stress is well known to alter the transcription and translation of specific genes and proteins responsible for governing mitochondrial functions and apoptosis. Scientific evidence advocates that the proliferator-activated receptor-gamma coactivator (PGC-1α) is vital for the optimal functioning of mitochondria and regulates the synthesis of mitochondrial complex subunits [25,26]. Alteration in respiratory chain complexes playsa pivotal role in initiating apoptosis inside living cells [27]. Evidence suggests that ACMP alters the Bax/Bcl-2 ratio and activates the caspase cascade, resulting in apoptosis [28]. A plethora of evidence has established that the advancement of the apoptotic cascade is closely connected to oxidative stress, thereby emphasizing the role of improving the mitochondrial antioxidant defense system [29,30]. Henceforth, the protective potential of natural antioxidants was sought to attenuate mitochondrial dysfunction where oxidative stress is implicated.

Berberine (BBR) is a natural alkaloid widely used as an effective therapeutic molecule owing to its antioxidant, anti-inflammatory, anti-apoptotic, and modulatory activities. The antioxidant properties can be attributed to metal chelation, free radical scavenging, and the potential to activate the endogenous antioxidant enzymes [31]. Berberine also improves mitochondrial integrity by increasing mitochondrial antioxidants and promoting mitochondrial biogenesis [32]. Moreover, studies have demonstrated the efficacy of BBR against mitochondrial anomalies in Alzheimer’s disease models [33] and mitochondrial dysfunction in high-fat diet-induced models [34]. BBR is also known to regulate the transcription of UCP-2 along with AMPk phosphorylation to restore insulin levels in diabetic mice [35].

The disruption of mitochondrial functions disturbs energy production and leads to the progression of various diseases. This study extends our earlier findings documenting the ameliorative effects of BBR against sub-chronic ACMP exposure-induced oxidative stress and inflammation in rat liver tissue [11]. It is well established that oxidative damage is closely linked with impaired oxidative phosphorylation, ultimately leading to mitochondrial dysfunction. A few studies have examined the oxidative stress of mitochondria in the testis and brain tissue of murine. However, there is a paucity of studies investigating the effects of ACMP on mitochondrial functions. Therefore, this study hypothesized to evaluate the toxic effects of ACMP exposure on mitochondrial functions and their amelioration by BBR in rats. More scientific studies and authentic data are needed on the molecular mechanism of mitochondrial dysfunction. In this study, we examined the ameliorative potential of BBR against ACMP-induced mitochondrial dysfunction and the role of PGC-1α signaling on mitochondrial antioxidant status (MnSOD and UCP-2), along with a transcription of mitochondrial subunits (ND1, ND2, COX1, and COX4) in liver tissue. We also evaluated the anti-apoptotic role of BBR in ACMP-exposed rat liver tissues, keeping in mind the fact that apoptosis is closely associated with oxidative stress and mitochondrial dysfunction.

## 2. Materials and Methods

### 2.1. Chemicals

Acetamiprid (#33674, C_10_H_11_ClN_4_), berberine (#B3251, C_20_H_18_ClNO_4_), bovine serum albumin (BSA) (#A3059), sucrose (#S9378), tris, ethylene diamine tetraacetic acid (EDTA) (#E-9884), 3,3′,5,5′-Tetramethylbenzidine (TMB) (#T0440), RNAlater (#R-0901), and sodium bicarbonate were purchased from Sigma-Aldrich (St Louis, MO, USA). Cytochrome c, NADH, glycyl glycine buffer, succinic acid, and potassium ferricyanide were attained from Sisco Research Laboratories (Mumbai, India). The nitrocellulose membrane (#GE10600002) was obtained from GE Healthcare (Freiburg, Germany). The primary antibodies of mouse anti-β-actin (#sc-47778), rabbit anti-caspase-3 (#sc-7148), rabbit anti-Bax (#sc-493), and rabbit anti-Bcl-2 (#sc-783) and secondary antibodies were procured from Santa-Cruz Biotechnology (Paso Robles, CA, USA). The RNA extraction kit (#NP-84105) and dNTP mix (#PGN018) were acquired from Genetix Biotech Asia (New Delhi, India). The cDNA synthesis kit (#K1621) and Taq polymerase (#EP0701) were obtained from Thermo Fisher Scientific (Waltham, MA, USA).

### 2.2. Animals and Their Care

Adult male albino rats (Wistar strain) of 150–180 g were acquired from the Disease Free Small Animal House of Lala Lajpat Rai University of Veterinary and Animal Sciences, Hisar. The rats were placed under standard laboratory conditions in polypropylene cages with free access to a standard pellet diet and water. The animals were acclimatized for 10 days before the start of the experiment. The Institutional Animal Ethical Committee duly approved the permission to use animals. All the protocols and animal handling were performed following the Committee for the Purpose of Control and Supervision of Experiments on Animals guidelines for the use and care of laboratory animals.

### 2.3. Experimental Design

Animals were randomly assigned to four groups, having at least six animals in each:

Group I (control) animals served as controls and received normal saline intragastrically via oral gavage.

Group II (BBR) animals were given 150 mg/kg b.wt BBR dissolved in saline (50 mg/mL) once a day by oral gavage for 21 consecutive days. Each animal received not more than 0.5 mL of BBR solution daily.

Group III (ACMP) rats intragastrically via oral gavage received 21.7 mg/kg b.wt ACMP for 21 days. Each animal received not more than 0.5 mL of ACMP solution daily.

Group IV (BBR+ACMP) animals received ACMP after 2 h of BBR administration (150 mg/kg b.wt) at a similar dose to the group II and III animals for 21 consecutive days.

After 24 h of completion of the last dose, the rats were euthanized under CO_2_ asphyxiation (6 L/min) as this procedure does not affect liver oxidative stress [36]. The liver tissue was removed, rinsed with ice-cold saline, and immediately used for mitochondria isolation, stored at −80 °C for western blot analysis, and a portion was dipped in RNALater for PCR analysis. A fraction of saline-free washed liver tissue was fixed in glutaraldehyde and stored in phosphate buffer for electron microscopic analysis.

### 2.4. Preparation of Mitochondria

The liver tissue was homogenized in 10 volumes of homogenizing buffer (pH 7.4) containing 0.25 M sucrose, 1 mM EDTA, and 5 mM tris using a glass Dounce homogenizer (Perfit, Ambala, India). The homogenate was centrifuged at 2100× *g* for 15 min at 4 °C to remove cellular debris. The supernatant was further centrifuged at 13,000× *g* for 15 min at 4 °C. The pellets were washed appropriately and redissolved in buffer and centrifuged at 3000× *g* for 5 min. The final obtained mitochondrial fraction was used for measuring complex activity after estimating the protein concentration followingthe method of Lowry et al. [37] using BSA as a standard. The activity of the mitochondrial complexes was assayed for five independent samples per group (n = 5) in duplicate.

### 2.5. Complex I Activity Assay

Complex I activity was assessed following the method of Kaur et al. [38]. This method involves NADH dehydrogenase-based oxidation to NAD^+^ and successive cytochrome C reduction. Briefly, the complex I activity was measured in 3 mL of reaction mixture containing 0.2 M glycyl glycine buffer (pH 8.5, 0.35 mL), 6 mM NADH (0.1 mL), 10.5 mM cytochrome C (0.1 mL), and 0.02 M sodium bicarbonate (20μL). The reaction was stimulated by adding 10 μL of mitochondrial sample and read at 550 nm using a spectrophotometer (Shimadzu UV-2450, Kyoto, Japan). The results were presented as nmol NADH oxidized/min/mg protein.

### 2.6. Complex II Activity Assay

Complex II activity was assessed based on the oxidation of succinate to fumarate by an artificial electron acceptor, i.e., potassium ferricyanide. Briefly, 25 μL of the mitochondrial sample was added to the mixture containing 0.2 M sodium phosphate buffer (pH 7.8, 1.5 mL), 0.6 M succinate (0.2 mL), 1% BSA (0.3 mL), and 0.03 M potassium ferricyanide (25 μL). The decrease in absorbance was measured spectrophotometrically at 420 nm, and the results were shown as nmol succinate oxidized/min/mg protein.

### 2.7. Complex IV Activity Assay

The activity of complex IV was assayed according to the method of Sandhir et al. [39] by determining the rate of oxidation of cytochrome C (reduced) at 550 nm. Briefly, a pinch of sodium borohydrate crystals was used to reduce cytochrome C and then adjusted to pH 7 with 0.1 M HCl. Finally, the reaction was initialized by adding 10 μL of mitochondrial sample to the mixture containing 0.1 mL reduced cytochrome c and 0.075 M sodium phosphate buffer (pH 7.4, 0.7 mL). The decrease in absorbance was read spectrophotometrically at 550 nm, and the values were presented as nmol cytochrome C oxidized/min/mg protein.

### 2.8. Semi-Quantitative PCR Analysis

The total RNA was isolated from the rat liver using an RNAsureMinikit according to the manufacturer’s instructions. The concentration and purity of RNA were quantified usinga Nanodrop spectrophotometer (Denovix, Wilmington, DE, USA). A total of 1 μg RNA was reversely transcripted to cDNA using a cDNA synthesis kit. GAPDH RNA (0.05 μg/μL) supplied in a cDNA synthesis kit was used as a control. cDNA was amplified using gene-specific primers retrieved from the previous studies [12,13,14] (Table 1) and β-actin as internal control on a gradient thermal cycler (PEQLAB, Erlangen, Germany) for 35 cycles consisting of 30 s each of denaturation (94 °C), annealing (30 s), and elongation (72 °C, 1 min). The final extension was carried out for 10 min at 72 °C. The resulting products were separated on agarose gel and visualized under a gel documentation system (XR+, Biorad Laboratories, Hercules, CA, USA). A gene-specific PCR analysis was carried out in three independent samples per group (n = 3) in triplicate.

### 2.9. Western Blotting

#### 2.9.1. Sample Preparation for Western Blotting

For total protein extraction, liver tissue was mixed with 10 volumes of homogenizing buffer and protease inhibitor. The tissue homogenate was blended by end-over-end inversion for 45 min and centrifuged at 2100× *g* for 15 min at 4 °C. The liver tissue lysate obtained was used for the analysis of apoptotic marker expression after estimating the protein concentration. A western blot analysis of each protein was performed in three independent protein samples per group (n = 3) in duplicate.

#### 2.9.2. Sodium Dodecyl-Sulfate Polyacrylamide Gel Electrophoresis (SDS-PAGE)

Liver tissue lysate containing 80–120μg of protein was diluted in Laemmli buffer (5X) (0.5 M tris (pH 6.8), 10% SDS, 10% glycerol, 0.01% bromophenol blue, and 5% β-mercaptoethanol). Medium-range molecular markers and samples were separated on 12% SDS-PAGE for 1 h 45 min. After electrophoresis, the proteins were transferred to the nitrocellulose membrane using a semi-dry blotting unit (Amersham Biosciences, Freiburg, Germany) at a voltage of 65 V for 45 min, followed by blocking in 5% skim milk for 1 h 30 min constant shaking at 12 shakes per min, to avoid non-specific binding. Then, the membrane was washed gently twice with PBS (10 shakes per min) and once with PBST (10 shakes per min) and was allowed to incubate overnight at 4 °C with specific primary antibodies (1:400 dilution in 2.5% skimmed milk) and then with appropriate HRP-conjugated secondary antibodies (1:2500 dilution in 2.5% skimmed milk) for 1h. The bands were developed using TMB for 4 to 8 min, and the band intensity was analyzed using Image J software (Version 1.54).

### 2.10. Electron Microscopy

Liver tissues of 1–2 mm^3^ were initially fixed in glutaraldehyde (2.5%) and stored in phosphate buffer and finally fixed in osmium tetraoxide. The fixed portions were dehydrated using graded ethanol and embedded in epoxy resin. The embedded portions were cut into thin sections (80 nm) using ultra-microtome and prepared for mounting. The prepared sections were mounted on the grid, stained, and examined under the transmission electron microscope (Tecnai TF-30, FEI-Thermo Fisher, Waltham, MA, USA) and captured images.

### 2.11. Statistical Analysis

The data were analyzed for normality of distribution and compared using a one-way analysis of variance (ANOVA) followed by Tukey’s post-hoc test using SPSS. The results were presented as mean ± SD. The *p* values ≤ 0.05 were considered significantly different.

## 3. Results

### 3.1. Complex I Activity Assay

**Figure 1:**
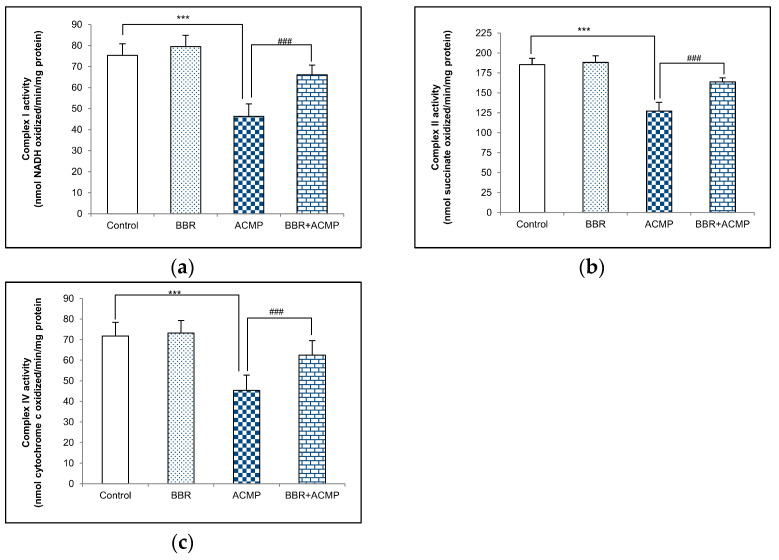
Effects of BBR supplementation and ACMP exposure on (**a**) complex I, (**b**) complex II, and (**c**) complex IV activity of mitochondria isolated from liver tissue of different experimental groups. Results are expressed as mean ± SD of 5 rats. *** is significant at *p* < 0.001 as compared with control rats. ^###^ is significant at *p* < 0.001 as compared with ACMP-exposed rats.

Complex I activity in rat liver mitochondria is shown in Figure 1a. A significant decrease in complex I activity by 39% was recorded in ACMP-exposed animals compared to controls. However, the activity was reinstated by 68% on BBR pre-treatment in BBR+ACMP co-treated rats. The activity of complex I was similar in control and BBR-treated groups.

### 3.2. Complex II Activity Assay

The activity of complex II was inhibited by 31% in ACMP-exposed rats’ livers compared to controls. However, the treatment of BBR in BBR+ACMP co-treated rats recovered the activity by 63%. Both control and BBR-treated rats showed similar complex II activity (Figure 1b).

### 3.3. Complex IV Activity Assay

Complex IV activity was significantly reduced by 37% in ACMP-exposed animals. Interestingly, prior supplementation of BBR in the BBR+ACMP co-treated group resulted in the restoration of complex IV activity by 65%. No significant changes were observed in the complex IV activity of control and BBR-treated animals (Figure 1c).

### 3.4. mRNA Expression of Mitochondrial Subunits

**Figure 2:**
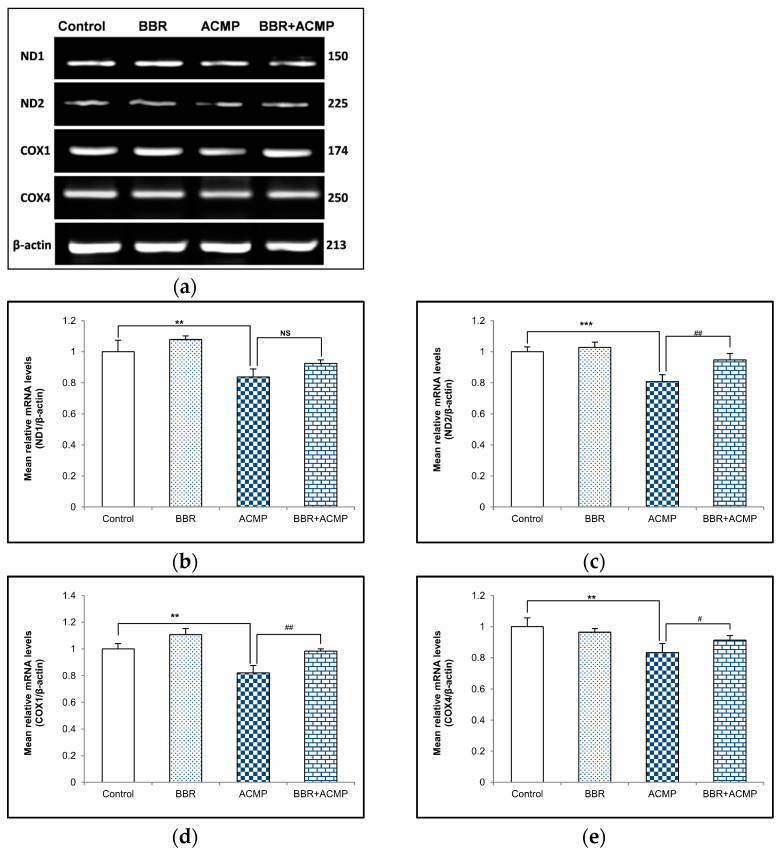
Effects of BBR supplementation and ACMP exposure on mRNA expression of mitochondrial complex subunits in liver tissue of different experimental groups. Mean relative mRNA level of (**b**) ND1, (**c**) ND2, (**d**) COX1, and (**e**) COX4 with respect to β-actin. Results are expressed as mean ± SD of 3 rats. ** is significant at *p* < 0.01 and *** is significant at *p* < 0.001 as compared with control rats. ^#^ is significant at *p* < 0.05, ^##^ is significant at *p* < 0.01, and NS is non-significant as compared with ACMP-exposed rats.

We performed a semi-quantitative PCR analysis of mitochondrial subunits to evaluate the effects of ACMP exposure at the mRNA level of these subunits. The mRNA expression of complex I subunits (ND1 and ND2) and complex IV subunits (COX1 and COX4) wassignificantly downregulated following ACMP intoxication compared to controls. Nevertheless, pre-treatment of BBR significantly improved ND1, ND2, COX1, and COX4 mRNA expression in BBR+ACMP co-treated animals compared to ACMP-administered rats. However, BBR-treated and control rats showed a similar mRNA expression to these subunits (Figure 2).

### 3.5. mRNA Expression of PGC-1α, MnSOD, and UCP-2

**Figure 3:**
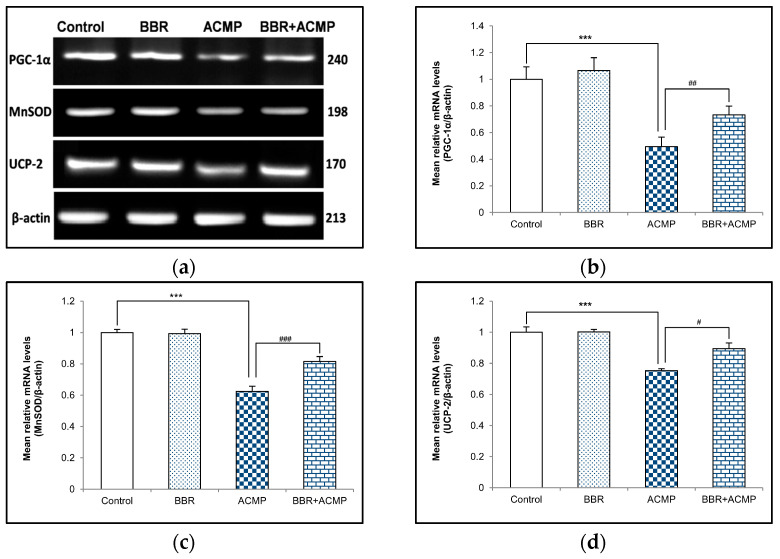
Effects of BBR supplementation and ACMP exposure on mRNA expression of mitochondrial antioxidants in liver tissue of different experimental groups. Mean relative mRNA levels of (**b**) PGC-1α, (**c**) MnSOD, and (**d**) UCP-2 were analyzed using densitometric analysis with respect to β-actin. Results are expressed as mean ± SD of 3 rats. *** is significant at *p* < 0.001 as compared with control rats. ^#^ is significant at *p* < 0.05, ^##^ is significant at *p* < 0.01, and ^###^ is significant at *p* < 0.001 as compared with ACMP-exposed rats.

To investigate the effect of ACMP exposure on PGC-1α and mitochondrial antioxidants, a semi-quantitative PCR investigation of PGC-1α, MnSOD, and UCP-2 was also performed. Acetamiprid exposure for 21 days resulted in 51%, 38%, and 25% downregulation in mRNA expression of PGC-1α, MnSOD, and UCP-2, respectively, in rat hepatic tissue compared to controls. However, pre-treatment of BBR in group IV upregulated mRNA expression of PGC-1α (48%), MnSOD (31%), and UCP-2 (19%) compared to ACMP-intoxicated rats. Rats treated with berberine alone showed no significant changes compared to controls (Figure 3).

### 3.6. Western Blotting Analysis

**Figure 4:**
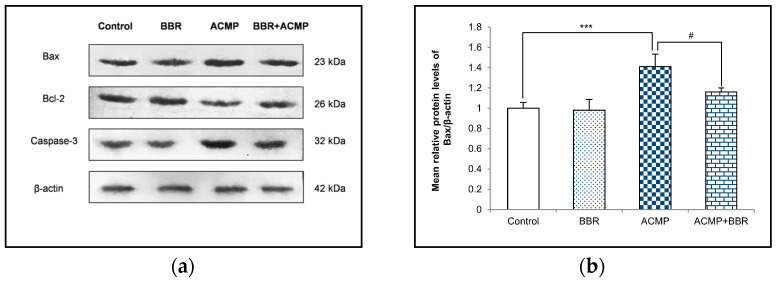

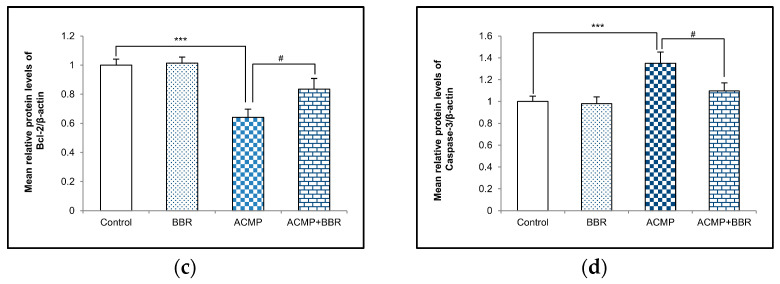
(**a**) Western blotting depicting the effects of BBR supplementation and ACMP exposure on apoptotic marker proteins in liver tissue of different experimental groups. Relative expression of (**b**) Bax, (**c**) Bcl-2, and (**d**) caspase-3 with respect to β-actin. Results are expressed as mean ± SD of 3 rats. *** is significant at *p* < 0.001 as compared with control rats. ^#^ is significant at *p* < 0.05 as compared with ACMP-exposed rats.

A western blot examination of Bax, Bcl-2, and caspase-3 was performed to elucidate the effects of ACMP intoxication on the protein level of apoptotic factors. A densitometric analysis showed a 36% decrease in the Bcl-2 protein level and an increase in the protein expression of Bax and caspase-3 by 41% and 35%, respectively, on ACMP exposure compared to controls. However, the pre-treatment of BBR in group IV significantly attenuated the increase in Bax and the caspase-3 level observed in ACMP-exposed rats by 61% and 72%, respectively, and replenished the Bcl-2 protein level by 54% compared to ACMP-administered animals. Both the control and the BBR treatments showed asimilar protein level of the apoptotic markers (Figure 4).

### 3.7. Electron Microscopic Examination

**Figure 5:**
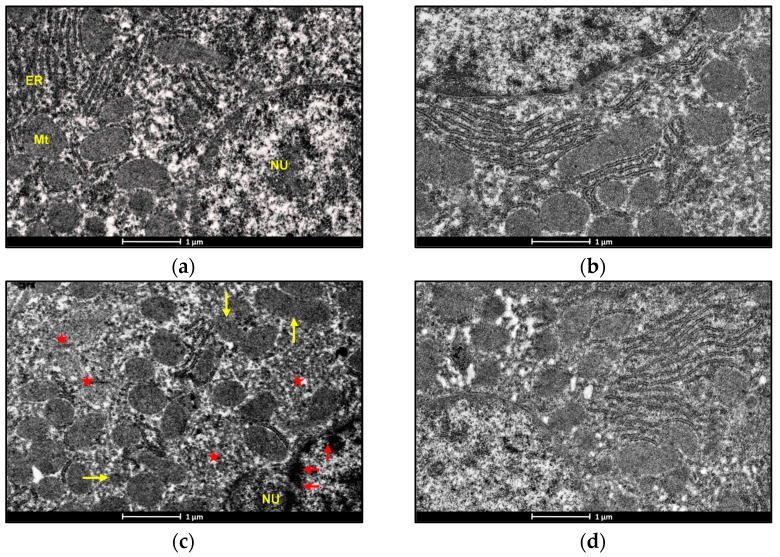
Photomicrographs representing the transmission electron microscopy of liver tissues of: (**a**) control groups; (**b**) BBR-treated group depicting nucleus (NU) normal distribution of mitochondria (Mt) and endoplasmic reticulum (ER); (**c**) ACMP-exposed group depicting chromatin condensation (red arrow), disruption of mitochondria (yellow arrow), loss of mitochondria (red star), and endoplasmic reticulum; and (**d**) BBR+ACMP co-treated group representing normal shape and distribution of mitochondria.

An electron microscopic examination of liver sections of control and BBR-treated rats represented the usual morphology of the nucleus and mitochondrion. Exposure to ACMP caused chromatin condensation, mitochondrial disruption, endoplasmic reticulum loss, and a reduced mitochondrial count in rat hepatocytes as compared to the control group (Figure 5a,c). Pre-administration of BBR to ACMP-exposed rats remarkably attenuated mitochondrial disruption, prevented chromatin condensation, and maintained mitochondrial and endoplasmic reticulum numbers compared to ACMP-intoxicated rats (Figure 5b–d).

## 4. Discussion

The administration of ACMP significantly reduced the enzymatic activities of complex I, II, and IV in rat liver mitochondria. Complex I is a prime rate-limiting enzyme complex that regulates oxidative phosphorylation, while complex II permits electron transfer. A decrease in complex IV activity suggests an oxidative imbalance and leads to mitochondria-dependent apoptosis. Thus, the decreased complex activity following ACMP exposure could lead to disruption of ETC and an increase in ROS generation, ultimately leading to impaired ATP production. On the other hand, prior supplementation of BBR to ACMP-exposed rats increased the activities of mitochondrial complexes that might be attributed to its antioxidant potential and free radical scavenging ability. Recently, we observed that BBR pre-treatment efficiently prevented mitochondrial dysfunction by restoring complex I, II, and IV activities in different rat brain regions [12]. Earlier, BBR was reported to efficiently prevent mitochondrial dysfunction via restoring mitochondrial complex activities in a transient global cerebral ischemia rat model [40]. The observed effects derived from the combined treatment of BBR with verapamil, which might have enhanced its uptake and showed synergistic action.

The decrease in complex activities might be associated with oxidative modifications of their subunits. Therefore, the mRNA expressions of complex I and complex IV subunits were assessed. The observed downregulation in transcriptional expression following ACMP exposure in ND1, ND2, COX1, and COX4 subunits is consistent with the decreased complex I and IV activities in rat liver tissue observed in the study. ACMP-mediated alterations in the mRNA expression of complex subunits might progress to the depletion of respiratory enzyme complexes. Moreover, BBR supplementation significantly regulated the transcription of mitochondrial complex subunits. Our results are in agreement with a recent study, wherein BBR supplementation was shown to restore the mRNA expression of complex I and IV activities in ACMP-exposed brain tissue of rats [12].

Impairment of respiratory chain enzymes might alter the antioxidant level inside mitochondria. PGC-1ɣ is a potential transcriptional activator and master modulator of mitochondrial biogenesis that plays a vital role in mitochondrial detoxification via regulating the expression of mitochondria antioxidants, including MnSOD and UCP-2 [25]. MnSOD is a metalloenzyme found in the mitochondria matrix that protects the cell against pathological conditions by superoxide dismutation, activating other transcription factors and inhibiting apoptosis. UCP-2 is another major mitochondrial antioxidant of the inner mitochondrial membrane that is crucial for cellular metabolism, proliferation, and cell death, thereby regulating mitochondrial oxidative damage [41]. The uncoupling of UCP-2 efficiently permits oxidative phosphorylation by inhibiting superoxide production and reducing ROS generation [42,43]. Thus, the effects of BBR treatment on the maintenance of depleted PGC-1α, MnSOD, and UCP-2 levels induced by ACMP were studied. The decreased mRNA expression of PGC-1α, MnSOD, and UCP-2 indicated ACMP-mediated oxidative stress and free radical generation inside mitochondria, leading to depletion of the antioxidant level. At the same time, pre-treatment of BBR to ACMP-exposed rats showed a significant increase in the mRNA expression of PGC-1α, MnSOD, and UCP-2, depicting its ameliorative effectiveness in reverting oxidative stress. The results suggested that the antioxidant activities of BBR might be responsible for improving the mitochondrial antioxidant system. These findings are supported by an earlier study, wherein BBR maintained mitochondrial antioxidants by inducing PGC-1α and MnSOD expression in doxorubicin-induced toxicity in the brain tissue of rats [44]. However, the post-transcriptional changes of these genes were not evaluated to reach a confirmation. Thus, gaining further insight into the role of BBR on these genes would be warranted to obtain concrete evidence.

Depletion in the antioxidant level and alterations in respiratory enzyme activity following ACMP exposure might lead to cellular injuries that elicit cell death signaling and apoptosis. Bcl-2 family proteins are potent regulators and consist of Bcl-2 and Bax. Bax is a pro-apoptotic protein whose increased expression indicates mitochondrial transition pore formation, eliciting increased membrane permeabilization. Bcl-2 is an anti-apoptotic protein that regulates the permeability of the mitochondrial membrane inside the cell. Caspase-3 is an executioner pro-apoptotic protein that initiates and regulates apoptosis inside the tissue. A growing body of evidence has demonstrated that ACMP exposure alters the Bax/Bcl-2 ratio and triggers the caspase cascade, resulting in apoptosis [26]. ACMP-mediated apoptosis might be ascribed to oxidative stress generation and stimulation of the mitochondrial apoptotic signaling pathway. The present study showed that ACMP exposure upregulated Bax and caspase-3 while it downregulated Bcl-2 expression, promoting the induction of apoptotic signaling in the hepatic tissue of rats. Conversely, supplementation of BBR pronouncedly attenuated ACMP-induced hepatic mitochondrial apoptosis by normalizing the expression of pro-apoptotic and anti-apoptotic markers. Earlier, various studies have demonstrated the anti-apoptotic potential of BBR in different models. Consistent with our results, Shaker et al. [44] showed the BBR-mediated inhibition of apoptosis via upregulating the Bcl-2 protein level and downregulating the Bax expression and Bax/Bcl-2 ratio in doxorubicin-induced cognitive impairment in rats. BBR pre-administration has also been reported to attenuate methyl–mercury chloride-mediated apoptosis in rat brains by reducing the Bax/Bcl-2 ratio and decreasing caspase-3 activity [45]. Similarly, BBR showed anti-apoptotic effects in the hippocampus of Alzheimer’s diabetic rats via regulating the caspase-cascade signaling pathway [46] and in the doxorubicin-intoxicated cerebral cortex of rats through downregulating caspase-3 expression [47].

Electron microscopic analysis is generally used to gain insight into the sub-cellular changes associated with exposure to xenobiotics, which are considered a serious contributor to organ toxicity. Through ROS production, oxidative changes in lipids and proteins, altering biochemical activities, and transcriptional changes, ACMP causes structural changes in cells and tissue [9,11,23]. In this study, ultra-structural changes in rat liver cells were observed, such as chromatin condensation and shrinkage, and the loss of mitochondria. The loss and disruption of mitochondria and chromatin condensation area direct result of ACMP exposure-induced mitochondrial dysfunction and apoptotic changes in rat hepatocytes. Meanwhile, the pre-treatment of BBR significantly prevented morphological changes and maintained mitochondrial integrity inside hepatocytes. The protective potential of BBR observed in this study is in accordance with the studies of Phogat et al. [12] reporting the reversal of ACMP-induced ultra-structural changes in rat brains. The evidence from the literature suggests that BBR shows protective potential against exposure to xenobiotics due to its functional groups, like methoxy and methylenedioxy [48]. Therefore, the findings of this study depict BBR as a potential hepatoprotective agent in terms of mitochondrial respiration, the antioxidant defense system, and ultrastructural alterations.

## 5. Conclusions

We evaluated the ameliorative potential of BBR, a natural alkaloid against ACMP exposure-induced mitochondrial dysfunction and apoptotic changes. We employed conventional biochemical insight, western blot, and electron microscopy, and explored transcriptional changes involved in the mitochondrial antioxidant defense system. This study is the first to reveal the potential therapeutic potential of BBR to attenuate mitochondrial oxidative stress, mitochondrial dysfunction, apoptosis, and cellular changes following ACMP exposure. Furthermore, BBR, with its efficacy in maintaining mitochondrial functions and apoptosis, holds great promise to be an effective and significant ameliorating agent against pesticides or chemical toxicity wherein mitochondrial functions are perturbed.

## Figures and Tables

**Table 1:** Sequence of specific primers used for semi-quantitative PCR analysis.

Primer	Accession	Primer Sequence(5′-3′)	Size (bp)
ND1	EU104724.1	F-TGGCCTTCCTCACCCTAGTA	150
		R-AGGTGGTTAGAGGGCGTATG	
ND2	EU104725.1	F-TCATCAGTCTTTGTTGGCGC	225
		R-TCATGCGAGTGAGAGTGTGT	
COX1	KF011917.1	F-CACATGAGCAAAAGCCCACT	174
		R-ACGGCCGTAAGTGAGATGAA	
COX4	NM_017202.1	F-GACTACCCCTTGCCTGATGT	250
		R-ACACGTAGCTCTTCTCCCAG	
PGC-1α	NM_031347.1	F-AGCCTCTTTGCCCAGATCTT	240
		R-GCAATCCGTCTTCATCCACC	
UCP-2	NM_019354.3	F-AGACCATTGCACGAGAGGAA	170
		R-AGAAGTGAAGTGGCAAGGGA	
MnSOD	NM_017051.2	F-ACAGGCCTTATTCCACTGCT	198
		R-CTACAAAACACCCACCACGG	
β-actin	V01217.1	F-TTGCCCTAGACTTCGAGCAA	213
		R-AGACTTACAGTGTGGCCTCC	

## Data Availability

All the data and materials associated with the findings stated in the results of this manuscript are within the manuscript.
